# Quantification of Urinary Phenyl-γ-Valerolactones and Related Valeric Acids in Human Urine on Consumption of Apples

**DOI:** 10.3390/metabo9110254

**Published:** 2019-10-29

**Authors:** Andrea Anesi, Pedro Mena, Achim Bub, Marynka Ulaszewska, Daniele Del Rio, Sabine E. Kulling, Fulvio Mattivi

**Affiliations:** 1 Department of Food Quality and Nutrition, Research and Innovation Centre, Fondazione Edmund Mach (FEM), 38010 San Michele all’Adige, Italy; andrea.anesi@fmach.it (A.A.); maria.ulaszewska@fmach.it (M.U.); 2 Human Nutrition Unit, Department of Food & Drug, University of Parma, 43121 Parma, Italy; pedromiguel.menaparreno@unipr.it; 3 Department of Physiology and Biochemistry of Nutrition, Max Rubner-Institut, 76131 Karlsruhe, Germany; achim.bub@mri.bund.de; 4 School of Advanced Studies on Food and Nutrition, and Microbiome Research Hub, University of Parma, 43121 Parma, Italy; daniele.delrio@unipr.it; 5 Human Nutrition Unit, Department of Veterinary Medicine, University of Parma, 43121 Parma, Italy; 6 Department of Safety and Quality of Fruit and Vegetables, Max Rubner-Institut, 76131 Karlsruhe, Germany; Sabine.Kulling@mri.bund.de; 7 Bioorganic Chemistry Laboratory, Department of Physics, University of Trento, 38123 Povo, Italy

**Keywords:** phenyl-γ-valerolactone, phenylvaleric acid, flavan-3ols, proanthocyanidins, apples, urine, LC-MS/MS, LC-MS, metabolic phenotype

## Abstract

Flavan-3-ols are dietary bioactive molecules that have beneficial effects on human health and reduce the risk of various diseases. Monomeric flavan-3-ols are rapidly absorbed in the small intestine and released in the blood stream as phase II conjugates. Polymeric flavan-3-ols are extensively metabolized by colonic gut microbiota into phenyl-γ-valerolactones and their related phenylvaleric acids. These molecules are the main circulating metabolites in humans after the ingestion of flavan-3-ol rich-products; nevertheless, they have received less attention and their role is not understood yet. Here, we describe the quantification of 8 phenyl-γ-valerolactones and 3 phenylvaleric acids in the urine of 11 subjects on consumption of apples by using UHPLC-ESI-Triple Quad-MS with pure reference compounds. Phenyl-γ-valerolactones, mainly as sulfate and glucuronic acid conjugates, reached maximum excretion between 6 and 12 after apple consumption, with a decline thereafter. Significant differences were detected in the cumulative excretion rates within subjects and in the ratio of dihydroxyphenyl-γ-valerolactone sulfate to glucuronide conjugates. This work observed for the first time the presence of two distinct metabotypes with regards to the excretion of phenyl-γ-valerolactone phase II conjugates.

## 1. Introduction

In the context of healthy lifestyles and disease prevention, flavan-3-ols are considered as dietary bioactive molecules, food constituents that are not essential for humans, but which may contribute in reducing the risk of various diseases and promoting healthy aging [1].

**Figure 1:**
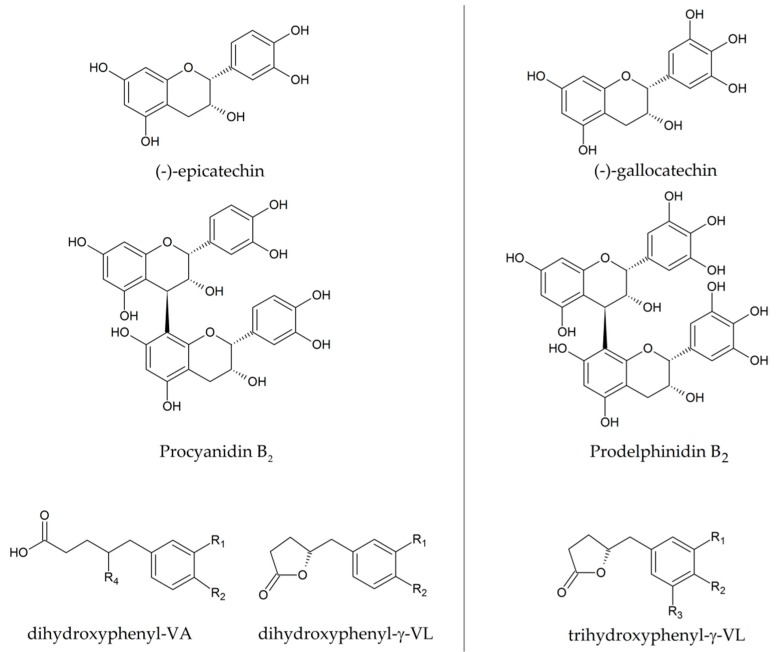
Left: structures of (‒)-epicatechin, the dimer procyanidin B_2_, a dihydroxyphenyl-γ-valerolactone (VL) and the related phenyl-valeric acid (VA). Right: structure of the (‒)-gallocatechin, its dimeric form prodelphinidin B_2_ and a trihydroxyphenyl-VL. Substituents (R_1_, R_2_, R_3_) can be hydroxyl groups, sulfate, methoxy, or glucuronic acid, while R_4_ can be hydrogen (yielding 5-(3’,4’-dihydroxyphenyl)VA) or hydroxyl group (yielding 4-hydroxy-5-(3’,4’-dihydroxyphenyl)VA).

Flavan-3-ols are a class of phytochemicals widely found in nuts, fruits, and vegetables, mostly in almonds, apples, banana, berries, pears, grapes and red wine, cocoa, and legumes [2,3,4,5,6,7,8]. Flavan-3-ols are found as simple monomers, (+)-catechin, (‒)-epicatechin, (‒)-epigallocatechin and their galloyl derivatives, or assembled into oligomers and polymers that are collectively named proanthocyanidins (PACs) (Figure 1).

Flavan-3-ols from cocoa have been extensively studied for their benefits on human health [7,9,10,11,12,13]. The consumption of cocoa flavan-3-ols proved to have cardioprotective effects in healthy individuals; improvement of endothelial function and flow-mediated dilation (FMD), decrease in blood pressure, increase in HDL, and decrease in LDL and lipid oxidation are among the most beneficial effects observed after regular consumption of cocoa. Positive effects were also demonstrated on the functionalities of the central nervous system, gastro-intestinal tract, and immune system [14]. 

In 2012, the Europe Community Regulation 1924/2006 authorized a health claim related to the assumption of cocoa flavan-3-ols to maintain good blood vessel elasticity: the daily consumption of 200 mg of cocoa flavan-3-ols is considered sufficient to obtain a beneficial effect [15].

The consumption of apples has proved to have similar positive effects on vascular function and has been positively correlated to a lower risk of lung cancer, abdominal aortic calcification, coronary heart disease, and type II diabetes [16,17,18]. The cardioprotective effects of apples have been attributed to the high content of flavan-3-ols, which improve endothelial function by enhancing nitric oxide (NO) bioavailability [18,19].

Apples are widely consumed worldwide and are a good source of flavan-3-ols [2,16,17,20,21]. Flavan-3-ols represent the major class of apple polyphenols, ranging between 71% and 90% of total polyphenols, depending on the cultivar [20]. The major constituents are oligomeric PAC (ranging from 38.8 mg to 162.2 mg/100 g of fresh weight, FW), procyanidin B2 (5.6–19.3 mg/100 g FW), and (‒)-epicatechin (5.2–18.4 mg/100 g FW), while (+)-catechin (0.5–3.40 mg/100 g FW) is present in lower amounts. Hydroxycinnamic acids (mainly 5′-caffeoylquinic acid and p-coumaroylquinic acid) represent between 4% and 18% of total polyphenols, flavonols (mainly quercetin glycosides) between 1% and 11%, dihydrochalcones (phloridzin and phloretin-xyloglucoside) between 2% and 6%, and anthocyanins (cyanidin glycosides in red apple varieties; 1–3%) are the other important constituents of apple polyphenols [20]. A remarkable diversity in the absolute content and pattern of the different polyphenols among apple species and cultivars has been evidenced, which is, in part, the result of the domestication process [21].

Monomeric flavan-3-ols are absorbed in the small intestine. Phase II enzymes transform them mainly into glucuronide-conjugates that can enter the systemic circulation, reaching a maximum plasma concentration within 2–4 h of food consumption [8,22,23,24]. In the liver, simple flavan-3-ols are converted to sulfate and methyl derivatives [4,25]. Dimeric and trimeric forms can also be absorbed, but to a lower extent and their bioavailability is up to 100-fold lower [24,26].

Oligomeric PAC with a degree of polymerization greater than *n* = 3 are not degraded in the stomach under acidic conditions and their polymeric nature limits the absorption in the small intestine. Therefore, their contribution to the pool of circulating monomeric flavan-3-ols in human plasma is neglectable [3,5,13,24,27]. It is estimated that over 70% of ingested flavan-3-ols passes un-metabolized through the upper part of intestine and reaches the colon, where PAC are extensively metabolized by local gut microbiota into phenyl-γ-valerolactones (PVLs) and their related phenylvaleric acids (PVAs) (Figure 1) [8,22,23,28]. PVLs have been proposed as (potential) biomarkers of flavan-3-ol intake [8,26]. Microbial flavan-3-ol catabolism is characterized by flavan-3-ol subunit cleavage (in the case of PACs), C-ring opening, lactonization, decarboxylation, dehydroxylation, and β-oxidation [3,5,25,29,30,31,32]. Phenylpropionic acids, phenylacetic acids, and benzoic acids are considered as end point catabolites of the microbial degradation of the flavonoid family. 

Microbial metabolites can be absorbed from the gut/colon and are then subjected to modifications in the liver by endogenous phase II enzymes; glucuronide-, sulfate- and methyl-conjugates enter the systemic circulation and are finally excreted in the urine, while non-conjugated metabolites are eliminated in the faeces [13,29,30,33].

Most of the data available on PVLs and PVAs is based on semi-quantitative, untargeted mass spectrometry (MS) [4,32,33], or targeted approaches lacking pure standards for accurate quantification [5,6,24]. With this approach, the annotation process is usually at level 2 or 3 of the Metabolomics Standards Initiative (MSI), i.e., with compounds putatively annotated (level 2) or putatively assigned to classes (level 3) and is lacking an accurate quantitation against a pure standard. Advances in asymmetric synthesis have led to the availability of pure PVL aglycones, glucuronidated, and sulfated conjugates, opening the way for robust quantitative and targeted-MS approaches [31,34] that can provide precious information on the bioavailability and nutrikinetics of flavan-3-ols in humans.

In this work, we report on the quantification of 8 PVLs and 3 PVAs in the urine of 11 subjects up to 48 h after apple consumption, using an UHPLC-ESI-Triple Quad-MS, together with the analysis of the phase II metabolites of epicatechin based on an untargeted approach. To the best of our knowledge, this is the first time that the accurate quantification of PVLs derived from apple flavan-3-ols is carried out.

## 2. Results

### 2.1. Flavan-3-ol and PAC Composition of Study Apples

Study participants ingested, on average, 688.85 μmol of (‒)-epicatechin, 176.35 μmol of procyanidin B1, 1882.18 μmol of procyanidin B2, and 2922.50 μmol of PACs, with a mean degree of polymerization (mDP) of 8.5 (Table 1). The complete composition of Elstar apples used for the intervention is available in Table S1. The two control treatments (placebo) lacked flavan-3-ols.

### 2.2. Targeted Analysis of Free and Conjugated PVLs and PVAs in Urine

The targeted metabolomics analysis of the urine collected revealed the presence of signals of 8 conjugated PVLs and 3 PVAs (Table 2). Free PVLs and PVAs were not detected. PVLs and PVAs were not detectable in urine samples from the control intervention (same diet exception of the apple intake).

Four of the screened analytes were unambiguously assigned to specific PVLs, according to the retention time of pure standards and mass spectra, and quantified with external calibration. Since external standards were not available for all the tested metabolites (e.g., methoxy-sulfate and methoxy-glucuronide and PVA), we opted to use chemically related molecules (see Table S2 for details).

In [32], 15 conjugated PVLs and 8 PVAs were found to be markers of apple juice intake, predominantly sulfate-, glucuronide-, and methoxy conjugates of dihydroxy-PVL and –PVA, or a combination thereof (i.e., methoxy-sulfate and methoxy-glucuronide conjugates). Taking into account the flavan-3-ol profile of the apple powder, the dihydroxy PVLs and PVAs identified could be mainly derivatives of 5-(3′,4′-dihydroxyphenyl)-γ-VL and 4-hydroxy-5-(3′,4′-dihydroxyphenyl)-VA, respectively [8]. 

The presence of glucuronide- and sulfate-conjugates of PVLs and PVAs have been confirmed in other studies after consumption of almond [6], cocoa [5,23], cranberry [8,35,36], grape [37], and green tea [28,29,38,39,40].

### 2.3. Bioavailability and Quantitative Determination of Conjugated PVL and PVA

Accurate determination of flavan-3-ol breakdown products is of great importance in order to study their metabolic fate. In the vision of a personalized nutrition, it is important to study the inter-individual differences and calculate the minimum amount necessary to obtain a health effect.

**Figure 2:**
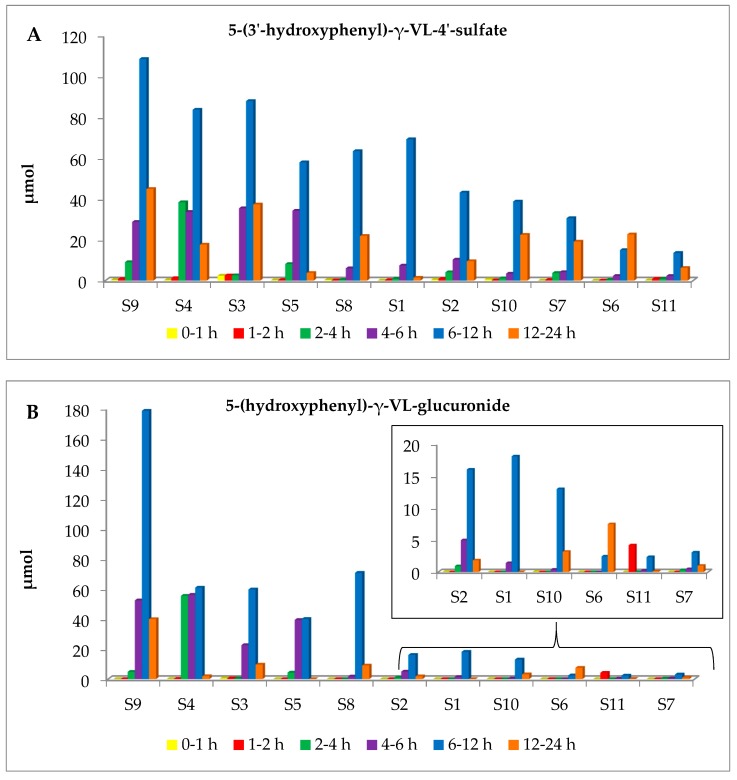
Urinary excretion of 5-(3′-hydroxyphenyl)-γ-VL-4′-sulfate (2) (**A**) and 5-(hydroxyphenyl)-γ-VL-glucuronide (4) (**B**) in the 11 study subjects (S1–S11) at different time intervals. 0–1 h: yellow bars; 1–2 h: red bars; 2–4 h: green bars; 4–6 h: purple bars; 6–12 h: blue bars; 12–24 h: orange bars. Subjects are shown in decreasing order of cumulative excretion in urine (AUC). Data were normalized according to urine volume.

Taking into account the most abundant metabolites (1, 2 and 4, which represent over 76.5% of the total excreted metabolites, all subjects but S6 exhibited maximum excretion between 6 h and 12 h, with a decline thereafter (Figure 2). Subject 6 reached maximum excretion between 12 h and 24 h. Subject 4 is the only one exhibiting notable excretion between 2 h and 4 h. Less abundant metabolites are not excreted by all participants. Quantitative data at each time interval are available in Table S3.

In the work of [32], PVLs did not reach their maximum plasma concentration in the first 5 h after apple juice consumption and the maximum concentration in urine was reached in the first 8–24 h. These deviations compared to the results presented here might be due to differences in the amount and type of flavan-3-ol intake. In human intervention studies on green tea, PVLs appeared in the bloodstream between 5 h and 12 h after green tea consumption, while renal extraction occurred between 7.5 h and 24 h [38,40,41]. Similarly, PVLs were detected in plasma within 6 h from cocoa intake and excreted in large amounts in urine up to 24 h [5].

From a quantitative point of view, there is a continuum in the cumulative excretion of conjugated PVLs and PVAs from high to low metabolizers (Figure 2). Apple intake resulted in cumulative excretion ranging from a minimum value of 70.68 μmol (S6) to a maximum of 524.35 μmol (S9); it is clear that significant differences, up to 7.5 times, exist within the subjects (Table 3).

Among the screened metabolites, 2 and 4 presented the largest increments in urine (cumulative excretion range 31.41–192.05 μmol and 4.82–275.60 μmol, respectively). Sulfate-conjugates of PVLs and PVAs were very abundant in this study, also in comparison to [32]. However, the use of appropriate reference compounds for the quantification in this work may provide more accurate quantitative data, overcoming one of the main limitations in the field of PVLs [31]. The percentage of excretion is also variable between subjects. Taking into account only the excretion relative to total PACs, this value ranges from 2.4% (S6) to 17.9% (S4).

### 2.4. The Ratio of Sulfate/Glucuronide Conjugates of 5-(3′,4′-dihydroxyphenyl)-γ-VL Suggests the Presence of Phase II Metabotypes 

Metabolites 2 and 4 represent the most abundant metabolites excreted in urine in all subjects, ranging from 71.0 (S6) to 91.2% (S5) of total metabolites, based on Area Under the Curve (AUC), except for S11. Methoxy-conjugates, sulfate-glucuronides, and PVAs were not considered in this calculation, as they represented a negligible proportion of the overall metabolites.

**Figure 3:**
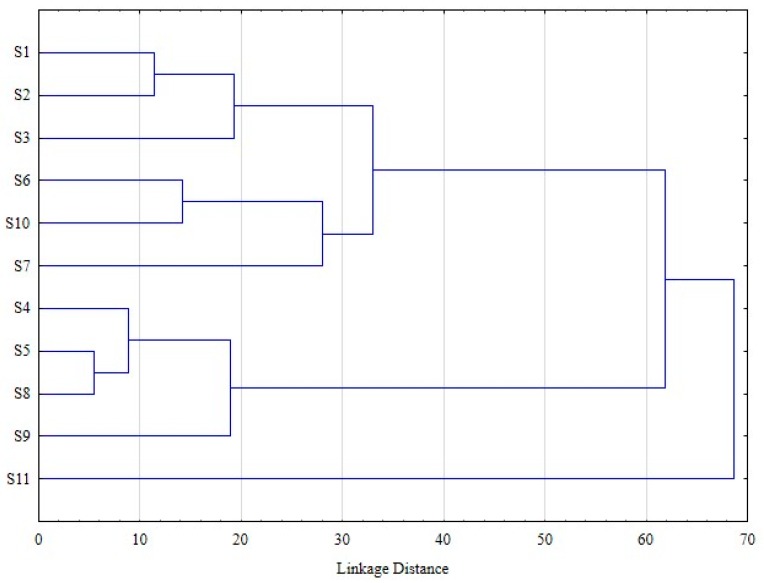
Cluster analysis based on percentages of excreted PVL and PVA metabolites in the 11 subjects (complete linkage, Euclidean distance).

The cluster analysis, based on all quantified metabolites, revealed the existence of two groups (S1, S2, S3, S6, S10, S7 and S4, S5, S8, S9), while S11 is singled out from all the other subjects (Figure 3). These differences are explicable, taking into consideration the ratio of AUC of metabolite 2 to AUC of metabolite 4, and are not dependent on subject metadata (age, gender, and BMI).

The first subpopulation (S1, S2, S3, S6, S10 and S7) displayed a ratio in favour of sulfate conjugates (>1.8), while the second one has a ratio of approximately 1 (Table 4). S11, despite having a ratio in favour of sulfate conjugates, has higher proportions of monohydroxyPVL (64.2%) compared to dihydroxyPVL (31.2%).

Subjects displaying a ratio in favour of sulfate-conjugates excreted lower amount of metabolites, except for S3. It is known that, for some individuals, the excretion is dependent on the substrates and can reach up to 50% of the ingested polyphenols [25]. Nevertheless, these differences, related to the existence of putative metabotypes on the phase II conjugation of dihydroxyPVLs, require further research by using a large number of subjects and 5-(3′-hydroxyphenyl)-γ-VL-4′-glucuronide or 5-(4′-hydroxyphenyl)-γ-VL-3′-glucuronide as standard for the quantification of metabolite 4, since here it was quantified as 5-(5′-hydroxyphenyl)-γ-VL-3′-glucuronide; the results might slightly change. 

### 2.5. Untargeted Analysis of Conjugated (–)-Epicatechin

**Figure 4:**
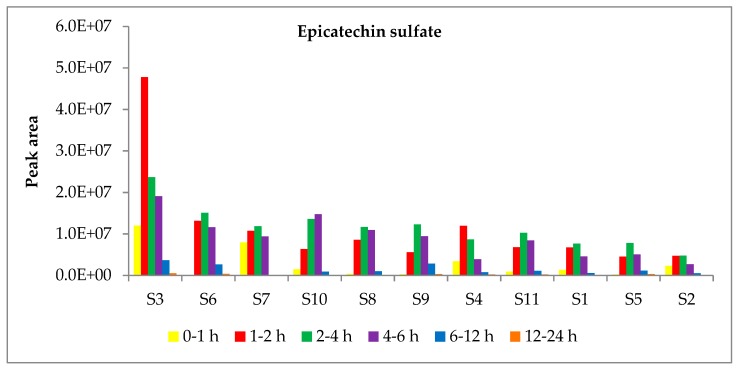
Excretion of epicatechin sulfate in urine in the 11 study participants (S1–11). 0–1 h: yellow bars; 1–2 h: red bars; 2–4 h: green bars; 4–6 h: purple bars; 6–12 h: blue bars; 12–24 h: orange bars. Subjects were shown in decreasing order according to the AUC in the range T1–T24.

The untargeted metabolomics approach revealed the presence of the sulfate, methoxy-sulfate, glucuronide, and methoxy-glucuronide conjugates of (–)-epicatechin in urine. The exact quantitation of epicatechin phase II metabolites was beyond the scope of this work, due to (a) the lack of commercially available standards for epicatechin phase II metabolites and (b) to avoid problems of misquantification derived from the quantification of epicatechin phase II metabolites according to (–)-epicatechin standard. However, as precursors of valerolactone and valeric acid derivatives, we reported their peak areas (see Table S4). As shown in Figure 4, epicatechin phase II metabolites are excreted in urine starting from 1 h to 6 h after apple consumption. The quantitative differences among the subjects in the urinary excretion of epicatechin conjugates were lower than those observed for PVLs and PVAs. In fact, excluding the high metabolizer S3, all the other subjects displayed similar cumulative excretion of epicatechin conjugates, with a fold change of 2.9 existing between S6 and S2 (data not shown). The cluster analysis based on epicatechin phase II metabolites was different than that obtained for PVL (Figure S1), suggesting that important differences exist between human and gut microbial metabolisms. The ratios epicatechin sulfate to glucuronic acid conjugates were also different from those obtained for PVL metabolites (Table 4).

## 3. Discussion

The biotransformation and bioavailability of monomeric flavan-3-ols has been the subject of many works. These metabolites, particularly (−)-epicatechin, are rapidly absorbed, modified, and released in the bloodstream within 1–4 h from food consumption and finally excreted in urine in the first 8–12 h after flavan-3-ol ingestion [8,22,23,28,45,46]. Our untargeted analysis demonstrated that (–)-epicatechin phase II metabolites are excreted in urine starting from 1 h to 6 h after apple consumption.

PVLs and PVAs are the main circulating metabolites in humans after ingestion of flavan-3-ol -rich food [5,8,30] and their appearance in the bloodstream and excretion in urine is delayed compared to that of monomeric flavan-3-ols, consistent with the microbial formation from flavan-3-ols. PVLs are excreted in amounts up to 10 times higher than (epi)catechin metabolites [22]; nevertheless, they have received less attention and their role is not totally understood yet. 

In [32], 15 conjugated PVLs and 8 PVAs were found to be markers of apple juice intake, predominantly sulfate-, glucuronide-, and methoxy conjugates of dihydroxy-PVL and –PVA or combination of thereof (i.e., methoxy-sulfate and methoxy-glucuronide conjugates). Taking into account the flavan-3-ol profile of the apple powder used in this study, the dihydroxy PVLs and PVAs identified could be mainly derivatives of 5-(3′,4′-dihydroxyphenyl)-γ-VL and 4-hydroxy-5-(3′,4′-dihydroxyphenyl)-VA, respectively [8]. 

The presence of glucuronide- and sulfate-conjugates of PVLs and PVAs have been confirmed in other studies after consumption of almond [6], cocoa [5,23], cranberry [8,35] in human, [36] in murine model, grape [37], and green tea [28,29,38,39,40].

The most recent literature sheds new light on the health effects connected with flavan-3-ol consumption and these microbial-derived metabolites are emerging as key metabolites [8]. However, there is the need for more data to extend the claims to other flavan-3-ol sources such as apples. In fact, the specific origin and type of flavan-3-ols is important in determining the health effect and safety profile of the bioactive compounds. For example, no adverse effects were reported for the 3-month consumption of 2000 mg/d of cocoa flavan-3-ols and procyanidins, while the 1-year consumption of 1315 mg of tea flavan-3-ols displayed adverse effects in post-menopausal women [13].

Two factors may influence the time and maximum PVL concentration in human: the degree of flavan-3-ol polymerization and the variation in the metabolic potential between individuals, which results from human genotype and the gut microbiota metabolic pathways.

The type and composition of parent flavan-3-ols determine the type of PVLs that can be biosynthesized; for example, trihydroxy-PVL can be produced only when gallo/epigallocatechin are present in the matrix, as in tea [39], and it is expected that these can be produced after the intake of any food or beverage containing prodelphinidins (such as wine) (see Figure 1 for molecular structures).

The Elstar apples used in this study did not contain notable amounts of gallo/epigallocatechin (0.004 μmol/apple of free epigallocatechin and 0.08 μmol/apple of epigallocatechin after phloroglucinolysis; data not shown), and trihydroxy-PVL was not detected. This is expected since apples, similarly to cocoa, only contain procyanidins [20].

The inter-individual variation in the production and excretion of PVLs and PVAs is known to be wide and different factors influence the absorption and metabolism of these compounds, mostly sex, age, dietary habits, and gut microbiota composition [8,39]. The composition of gut microbiota is the most important factor in defining the inter-individual variability in the production of different PVL and PVA aglycones (mono-, di- or trihydroxy-PVL and PVA). In a study by the authors of [42], it was postulated that the production of dihydroxy-PVLs depends on gut microbiota composition rather than the specific food matrix. The presence of bacteria belonging to *Clostridia*, *Actinobacteria*, and *Propionabacteria* genera was positively correlated with this capacity. In a work by the authors of [32], the correlation between PVL and PVA plasma and urinary metabolic profiles with gut microbiota composition revealed that a high production of these metabolites is positively associated with the presence of *Dialister*, *Prevotella,* and *Escherichia* genera and negatively associated with *Anaerostipes*, *Turicibacter*, *Lachnospiracaea incertae sedis*, *Coprococcus*, and *Blautia* in faeces.

Differences in the polymorphism of phase II enzymes also contribute to individual variability in circulating metabolites. In humans, 15 isoforms of UDP-glucuronosyltransferases have been identified and they display wide polymorphic expression patterns, which could explain the high inter-individual variability in glucuronidation of polyphenols [3,43]. The sulfonation pathway has higher-affinity than the glucuronidation one but lower capacity, so that when the ingested flavan-3-ol dose increases, a shift from sulfonation toward glucuronidation may occur [44]. This balance is also affected by species, sex, and food deprivation [3].

The existence of metabotypes (also named metabolic phenotypes) has been to date demonstrated for the formation of 8-prenylnaringenin from hop prenylflavonoids [45], equol from soy daidzein [46], urolithins from walnut and pomegranate ellagitannins [47], and PVLs and 3-(hydroxyphenyl)-propionic acid from green tea flavan-3-ols [39]. All these metabolites are microbially formed and the existence of metabotypes can be attributed to differences in the composition of the gut microbiota. 

Here, we propose for the first time the presence of different metabotypes with regards to the excretion of dihydroxyPVL phase II conjugates after apple consumption. As our study is conducted with a limited number of subjects, further investigations on a wide number of individuals and repeated time points could shed light on this finding and confirm or reject the hypothesis of the presence of stable metabotypes. Furthermore, detailed information on UDP-glucuronosyltransferase as well as sulfotransferase isoforms involved in the conjugation of dihydroxyPVL is also needed to unravel underlying mechanisms. However, it appears that the variance in the profile of gut microbiota-derived metabolites is higher compared to that of host-derived metabolites, as demonstrated by the fold change differences among subjects (7.9 for PVL and PVA, 2.9 for phase II metabolites of epicatechin).

Conventional wisdom says, “An apple a day keeps the doctor away”. However, PVLs and PVAs are likely to be important effectors in determining the beneficial effects of apple consumption. Our results demonstrated that these metabolites, chemically equal to those produced by gut microbiota after ingestion of almond, cocoa, cranberry, red grape and wine, and green tea, are persistent in the human body in consistent amounts up to 24 h after apple consumption. 

It is estimated that the consumption of a single standard serving of apple in Italy (150 g) can provide, on average, 101.9 mg of flavan-3-ols [20]. From a bioequivalence view, the consumption of two apples per day could provide the same amount of flavonols as that reported in EFSA NDA panel for cocoa (200 mg) [15], therefore, exerting beneficial effects for human health. It is expected that if any of these metabolites are involved in the healthy properties of the apples, the presence of a huge diversity in the capacity to produce these circulating metabolites among subjects should impact the clinical outcome of the study. We suggest that the application of our protocol, providing the intake of a controlled amount of flavan-3-ols followed by the analysis of the 24-h urine, could lead to the selection of groups of participants with similar metabolic capacity, and the subsequent diminution of a major source of variation within clinical studies. The approach could help to define the protective role of dietary flavan-3-ols and their catabolites.

## 4. Materials and Methods 

### 4.1. Reagents and Chemicals

Acetonitrile UHPLC-grade and formic acid-MS grade were purchased from Sigma-Aldrich (St. Louis, MO, USA). Ultrapure Milli-Q deionized water was obtained from Elix (Merck-Millipore, Milan, Italy). 5-phenyl-γ-valerolactone-3′-sulfate, 5-phenyl-γ-valerolactone-3′-glucuronide, 5-(5′-hydroxyphenyl)- γ-valerolactone-3′-sulfate, 5-(3′-hydroxyphenyl)-γ-valerolactone-4′-sulfate, and 5-(5′-hydroxyphenyl) -γ-valerolactone-3′-glucuronide were synthesized in house [31,34]. The nomenclature used follows the rules proposed by [8]. Most of these molecules are catalogued on the standards sharing platform FoodComEx (www.foodcomex.org).

### 4.2. Dietary Intervention and Sample Collection

This randomized, controlled crossover study was performed at the Division of Human Studies of the Max Rubner-Institut in Karlsruhe, Germany, in January to March 2016, in accordance with the Declaration of Helsinki, approved by the Ethics Committee of the State Medical Chamber Baden-Wuerttemberg (F-2015-101) and registered at the German Clinical Trials Register (DRKS00008787; UTN U-1111-1177-1536). A total of 6 healthy men and 6 women were recruited by advertisements in local media and the MRI study participant data base. Inclusion criteria were age 18–40 years and body mass index (BMI) 18.5–30 kg/m^2^. Exclusion criteria were diseases related to digestion, metabolism or excretion of nutrients, known allergies to the intervention food, supplement use within 4 weeks, regular medication except hormonal contraceptives, use of antibiotics within 6 months, pregnancy, lactation, blood donation within 3 months, smoking, and unwillingness to follow dietary restrictions. Eligibility was assessed after taking medical history and physical examination and volunteers provided written informed consent.

**Figure 5:**
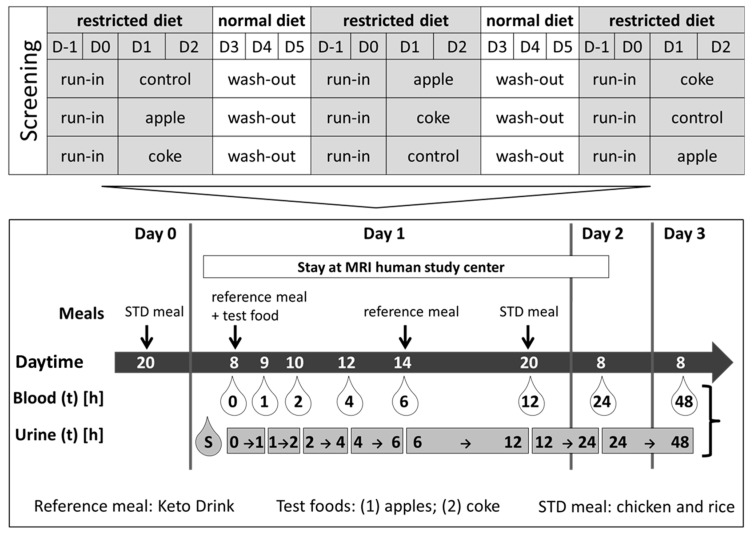
Study design of the randomized, controlled crossover study.

Each volunteer was assigned to three different test products in random order: 1) 500 mL of water as control with 200 mL of Keto-Drink (TARVALIN AG, Germany) (CT), 2) 400 g of apples (var. Elstar), cut in slices without apple core) with 200 mL of Keto-Drink (AP), and 3) 500 mL of soft drink (Coca Cola^®^) with 200 mL of Keto-Drink (SD) (Figure 5). The Keto-Drink, which was chosen because of its very low sugar content, was purchased from a pharmacy; a detailed composition of the Keto-Drink is given in Table S5; apples and soft drink were from local suppliers. The study arm with the soft drink has no significance for the research question addressed here and was therefore not evaluated in this work.

In the morning of the experimental day (Day 2), fasted subjects entered the study center, provided spot urine, and a cannula (Venofix Safety, Braun, Melsungen, Germany) was inserted into an antecubital vein. They stayed overnight until the next morning. Blood was drawn in supine position in S-Monovette tubes (Sarstedt AG, Nümbrecht, Germany) before and 1, 2, 4, 6, 12, and 24 h, respectively, after intake of the test foods. The test foods were consumed within 15 min. Urine samples were concomitantly collected in plastic containers at 0–1 h (T1), 1–2 h (T2), 2–4 h (T4), 4–6 h (T6), 6–12 h (T12), and 12–24 h (T24). Additionally, urine was collected by volunteers at home for the following 24 h (day 3; 24–48 h), stored in provided cooler bags, and immediately brought to the study center. In the evening of Day 1 (run in) and at Day 2 after 12 h, the participants received a standard meal ad libitum (steamed chicken with boiled rice, butter, salt). In addition, at day 2 (6 h), at least 200 mL of Keto-Drink was provided. The participants were instructed to refrain from fruit and fruit-containing food, as well as sugar-rich beverages and foods during the entire experiment (day 0 to day 3). Interventions were separated by a 6-day washout period. 

Urine was stored at 4 °C between collections, and the volume of each interval collection was recorded before preparing aliquots. Urine samples were centrifuged at 1850 g (4 °C, 10 min) to remove cellular particles and debris. Before processing, all urine samples were checked with urine test strips (CombiScreen, CombiScan 100, Analyticon) to exclude samples with pathological aspects and stored at −80 °C without preservatives until analysis. Here, we report urinary data from the apple and control intervention part of the study for 6 male and 5 female participants. One female volunteer was excluded from the analysis due to menstrual blood contamination in the urine samples.

### 4.3. Sample Preparation for PVL Quantitation

Urine samples were thawed on ice and vortexed for 10 s. 40 μL urine were diluted with 160 μL of 0.1% formic acid in water (1:5; *v*/*v*); the samples were centrifuged at 18,000× *g* for 5 min and then placed into sealed MS-vial with a 250 μL glass insert. Quality control sample was created by pooling 40 μL of each sample.

### 4.4. Targeted UHPLC-ESI-QqQ-MS

The method for PVL quantitation was developed and validated by [31]. Briefly, chromatographic separation was performed on a Kinetek EVO C18 (100 × 2.1 mm I.D., 2.6 μm particle size) purchased from Phenomenex (Phenomenex, Bologna, Italy). Mobile phase A was water containing 0.2% formic acid, mobile phase B was acetonitrile with 0.2% formic acid. The gradient was slightly modified as follows: 5% B was maintained for 0.5 min, then 25% B was reached in 1.5 min and maintained for 2 min; then 50% B was reached in 2 min and finally 95% B was reached in 1 min and kept for 1 min. Column was re-equilibrated at starting conditions for 4 min. The flow rate was 0.4 mL/min, the injection volume was 5 μL, and the column oven was set at 40 °C. Sample tray temperature was 10 °C.

The detection was performed on a Waters ^®^ Xevo QqQ–MS equipped with ESI source (Waters, Milan, Italy). The MS operated in negative ion mode; the capillary was set at 270 °C and the source at 300 °C; source voltage was 3 kV. Ultra-high purity argon was used as collision gas. Characteristic MS conditions were optimized by infusing pure PVLs according to 31. The injection sequence was randomized with regards to study participants and treatments. Quality control samples were acquired at the beginning of the sequence in order to condition the column and stabilize the MS response and were injected every 10 samples in order to address the stability of our measurements.

Calibration curves of standard PVL were prepared by serial dilution and acquired in the range 100 μM–0.1 nM. PVAs were quantified by using calibration curves of chemically related PVLs. Results of calibration are reported in Table S1. Data processing was performed using MassLynx V4.1 software (Waters, Milan, Italy).

### 4.5. Sample Preparation for Untargeted Analysis and Targeted Selection of Epicatechin Phase II Metabolites

100 µL of urine were placed into 96-well plate Millipore PVDF and 100 µL of internal standards in methanol were added (hyppuric acid-d5 and tryptophan-d5). The mixture was filtered using a positive pressure-96 manifold. Then, 300 µL of external standard in MilliQ water (trans-cinnamic acid-d_5_, cholic acid-d_4_, octanoyl-L-carnitine-d_3_) were added to the collection plate. Such prepared urine samples were submitted to the LC-LTQ-XL-Orbitrap-MS analysis. Protocol of instrumental analysis was described elsewhere [48]. Data processing protocol was published previously [33]. Briefly, raw data files were converted to mzXML format with the msconvert utility included in ProteoWizard [49]. Profiling data was processed with XCMS [50] using the ‘‘matchedFilter’’ peak picking method. A signal-to-noise ratio cut off of 4 was used, full width at half maximum was set to 10s, and a step size of 0.005 Da was used for extracting chromatograms. The resulting feature matrix was annotated using CAMERA [51] to group features corresponding to the same parent ion species. Results from the untargeted experiments will be published in a separate article.

We browsed this dataset and specifically targeted the *m/z* features of (epi)catechin metabolites according to our previous investigation that was focused on apple intake biomarker discovery [32]: (epi)catechin sulfate (*m/z* 369.0279, neg), (epi)catechin glucuronide (*m/z* 465.1002, neg), (epi)catechin methoxy sulfate (*m/z* 383.0416, neg), and (epi)catechin methoxy glucuronide (*m/z* 481.1337, pos). Mass spectrometry details are reported in Table S6.

### 4.6. Determination of Flavan-3-Ols Content in Study Apples

Elstar apples (approximately 1 kg) were cut and cores with seeds were removed. Apples were freeze-dried for 4 days and subsequently ground with a knife mill 3 times for 20 s each at 8000 rpm (Grindomix GM200; Retsch, Haan, Germany). The powder was stored at −80 °C until analysis.

Samples of freeze-dried apples were analysed in triplicate, as described in [20]. 1 g of powder was diluted with 0.5 mL of methanol including rosmarinic acid as internal standard (4 ppm). The sample was centrifuged 10 min, 15,000 rpm at 4 °C and the supernatant was filtered with 0.22 µm, 13 mm Millex-GV PDVF filters (Millipore, Burlington, MA, USA). The phenolic extract was analysed with Waters^®^ UHPLC-ESI-Xevo Triple Quadrupole –MS (Waters^®^, Milford, MA, USA). The content of PACs and their mean degree of polymerization (mDP) was determined by UHPLC-DAD-QqQ-MS before and after phloroglucinolysis, according to validated method protocols [52].

### 4.7. Statistical Analyses

Relative abundance of each metabolite was calculated as percentage respect to each subject cumulative excretion. For low abundant metabolites, zero values were replaced with one-tenth of the minimum value quantifiable with calibration curves. Cluster analysis (complete linkage, Euclidean linkage) was performed with Statistica v 13.3 (TIBCO Software Inc., Palo Alto, CA, USA).

## Figures and Tables

**Table 1:** Estimated content of monomeric flavan-3-ols and dimeric and oligomeric PAC in the apple var. Elstar (*n* = 3)**.**

Flavan-3-ol	Content (μmol)
Free (+)-catechin	86.57 ± 4.68
Free (−)-epicatechin	688.85 ± 29.96
Free gallocatechin	0.17 ± 0.03
Free epigallocatechin	0.00 ± 0.00
Free catechin gallate	0.18 ± 0.00
Procyanidin B1	176.35 ± 11.90
Procyanidin B2	1882.18 ± 62.13
Total PACs	2922.50 ± 121.74
PAC mDP	8.5 ± 0.01
Total flavan-3-ols	5756.81

**Table 2:** Conjugated PVL and PVA detected in the urine of 11 study participants with the transition used for their quantitation and their retention times. VL: valerolactone; VA: valeric acid.

Metabolite	ID	Transition (*m/z*)	Retention Time (min)
5-phenyl-γ-VL-3′-sulfate	1	271 > 191	2.88
5-(3′-hydroxyphenyl)-γ-VL-4′-sulfate	2	287 > 207	2.66
5-phenyl-γ-VL-3′-glucuronide	3	367 > 191	2.10
5-(hydroxyphenyl)-γ-VL-glucuronide (3 ′,4 ′ isomer)	4	383 > 207	1.76
5-phenyl-γ-VL-methoxy-sulfate 1	5	301 > 206	2.65
5-phenyl-γ-VL-methoxy-sulfate 2	6	301 > 206	2.83
5-phenyl-γ-VL-sulfate-glucuronide	7	463 > 207	2.05
5-(phenyl)-γ-VL-methoxy-glucuronide	8	397 > 221	2.03
4-hydroxy-5-(hydroxyphenyl)-VA-sulfate	9	305 > 225	2.16
4-hydroxy-5-(hydroxyphenyl)-VA-glucuronide	10	401 > 225	2.05
4-hydroxy-5-phenyl-VA-methoxy-sulfate	11	319 > 239	2.12

**Table 3:** Urine cumulative excretion of phenyl-γ-valerolactones and phenylvaleric acids (μmol) in the interval T1–T24 in 11 subjects (S1–11). The values reported in brackets indicate the percentage of each metabolite on the sum of all detected compounds. ACE: average cumulative excretion (± standard deviation).

PVL and PVA Metabolites	Cumulative Excretion (μmol) and Relative % of Different Metabolites in the 11 Study Subjects											
	S9	S4	S3	S5	S8	S2	S1	S10	S6	S11	S7	ACE
**5-phenyl-γ-VL-3′-sulfate**	8.61 (1.6)	10.85 (2.7)	2.43 0.8)	2.95 (1.4)	0.56 (0.3)	8.33 (7.7)	2.17 (1.9)	24.09 (20.9)	7.02 (9.9)	53.23 (43.3)	3.31 (4.5)	11.23 (±15.38)
**5-(3′-hydroxyphenyl)-γ-VL-4′-sulfate**	192.05 (36.6)	174.55 (42.9)	168.04 (58.1)	104.29 (50.6)	91.97 (47.6)	68.18 (62.7)	79.06 (70.1)	66.24 (57.58)	40.21 (56.9)	31.41 (25.6)	57.79 (77.8)	96.92 (±56.92)
**5-phenyl-γ-VL-3′-glucuronide**	6.27 (1.2)	4.26 (1.0)	0.03 (0.0)	0.42 (0.2)	0.04 (0.0)	0.04 (0.0)	0.05 (0.0)	2.76 (2.4)	3.70 (5.2)	25.60 (20.8)	0.04 (0.1)	3.93 (±7.51)
**5-(hydroxyphenyl)-γ-VL-glucuronide**	275.60 (52.6)	174.40 (42.9)	93.17 (32.2)	83.63 (40.6)	81.44 (42.2)	23.81 (21.9)	19.54 (17.3)	16.61 (14.4)	9.93 (14.1)	6.91 (5.6)	4.82 (6.5)	71.81 (±85.64)
**5-phenyl-γ-VL-methoxy-sulfate (1)**	0.44 (0.1)	0.52 (0.1)	0.46 (0.2)	0.39 (0.2)	0.36 (0.2)	0.34 (0.3)	0.31 (0.3)	0.34 (0.3)	0.23 (0.3)	0.39 (0.3)	0.29 (0.4)	0.37 (±0.08)
**5-phenyl-γ-VL-methoxy-sulfate (2)**	1.30 (0.2)	1.00 (0.2)	1.11 (0.4)	0.84 (0.4)	0.57 (0.3)	0.59 (0.5)	0.59 (0.5)	0.50 (0.4)	0.33 (0.5)	0.36 (0.3)	0.33 (0.4)	0.68 (±0.33)
**5-phenyl-γ-VL-methoxy-glucuronide**	1.96 (0.4)	1.18 (0.3)	0.80 (0.3)	0.81 (0.4)	1.07 (0.6)	0.62 (0.6)	0.81 (0.7)	1.40 (1.2)	0.30 (0.4)	0.43 (0.6)	0.39 (0.5)	0.89 (±0.49)
**5-phenyl-γ-VL-sulfate-glucuronide**	30.91 (5.9)	33.92 (8.3)	12.30 (4.3)	10.67 (5.2)	9.57 (5.0)	6.83 (6.3)	5.51 (4.9)	3.10 (2.7)	6.87 (9.7)	3.20 (2.6)	5.49 (7.4)	11.67 (±10.67)
**4-hydroxy-5-(hydroxyphenyl)-VA-sulfate**	7.18 (1.4)	6.14 (1.5)	10.60 (3.7)	2.07 (1.0)	7.39 (3.8)	<LOD (0.0)	4.67 (4.1)	0.06 (0.1)	1.85 (2.6)	0.30 (0.2)	1.82 (2.4)	3.82 (±3.58)
**4-hydroxy-5-(hydroxyphenyl)-VA-methoxy-sulfate**	0.02 (0.0)	0.01 (0.0)	0.04 (0.0)	0.01 (0.0)	0.02 (0.0)	<LOD (0.0)	<LOD (0.0)	<LOD (0.0)	0.02 (0.0)	0.01 (0.0)	0.01 (0.0)	0.01 (±0.01)
**4-hydroxy-5-(hydroxyphenyl)-VA-glucuronide**	0.02 (0.0)	0.17 (0.0)	0.21 (0.1)	0.02 (0.0)	0.04 (0.0)	0.02 (0.0)	0.03 (0.0)	0.02 (0.0)	0.21 (0.3)	0.98 (0.8)	0.02 (0.0)	0.16 (±0.28)
**SUM (μmol)**	524.35	406.98	289.18	206.10	193.03	108.77	112.73	115.13	70.68	122.79	74.30	202.19 (±147.53)
**% excretion (to all flavan-3-ols)**	9.1	7.1	5.0	3.6	3.4	1.9	2.0	2.0	1.2	2.1	1.3	3.52 (±2.56)
**% excretion (to total PACs)**	17.9	13.9	9.9	7.0	6.6	3.7	3.9	3.9	2.4	4.2	2.5	6.90 (±5.04)

**Table 4:** Ratio AUC (dihydroxy-PVL-sulfate/glucuronide), total AUC, and ratio epicatechin sulfate/glucuronide in the 11 study participants.

Subject	Ratio 5-Hydroxyphenyl-γ-VL Sulfate/Glucuronide	Cumulative Excretion (μmol)	Ratio Epicatechin Sulfate/Glucuronide
S1	4.0	112.73	3.7
S2	2.9	108.77	7.2
S3	1.8	289.18	6.8
S4	1.0	406.98	5.8
S5	1.2	206.10	6.7
S6	4.0	70.68	3.9
S7	12.0	74.30	8.9
S8	1.1	193.03	2.5
S9	0.7	524.35	2.3
S10	4.0	115.13	3.6
S11	4.5	122.79	4.8

## Supplementary Materials

The following are available online at https://www.mdpi.com/2218-1989/9/11/254/s1, Table S1: Flavan-3-ol composition of study apples as determined by UHPLC-ESI-QqQ-MS/DAD; Table S2: Standard compounds used for PVL and PVA quantitation, their retention times, linearity range, curve equation, and R2; Table S3: Quantification of selected PVLs and PVAs at different time points in urine of 11 study subjects; Table S4: Peak intensities of epicatechin phase II metabolites at different time points in urine of 11 study participants; Table S5: Composition of the Keto-Drink; Table S6: Mass spectrometry characteristic of (epi)catechin metabolites in urine samples after apple intake; Figure S1: Cluster analysis based on percentages of excreted epicatechin phase II metabolites in the 11 subjects (complete linkage, Euclidean distance). Metabolomics data have been deposited to the EMBL-EBI MetaboLights database [53] with the identifier MTBLS1313. The complete dataset can be accessed here https://www.ebi.ac.uk/metabolights/MTBLS1313.
