# Supplementary material for: Quantification of Urinary Phenyl-γ-Valerolactones and Related Valeric Acids in Human Urine on Consumption of Apples
Source: Metabolites. 2019 Oct 29;9(11):254. doi: 10.3390/metabo9110254 (PMC6918130; doi:10.3390/metabo9110254)
Supplement: Supplementary file 1 [file metabolites-09-00254-s001.pdf]

Quantification of urinary phenyl-γ-valerolactones and related valeric acids in human urine after consumption of apples.

Andrea Anesi [1], Pedro Mena [2], Achim Bub [3], Marynka M. Ulaszewska [1], Daniele Del Rio [4,5,] Sabine E. Kulling [6] and Fulvio Mattivi [1,7]

[1] Department of Food Quality and Nutrition, Research and Innovation Centre, Fondazione Edmund Mach (FEM), Via Mach 1, 38010 San Michele all 'Adige, TN, Italy;
[2] Human Nutrition Unit, Department of Food & Drug, University of Parma, Parma, Italy
[3] Department of Physiology and Biochemistry of Nutrition, Max Rubner-Institut, Karlsruhe, Germany
[4] School of Advanced Studies on Food and Nutrition, and Microbiome Research Hub, University of Parma, Parma, Italy
[5] Human Nutrition Unit, Department of Veterinary Medicine, University of Parma, Parma, Italy
[6] Department of Safety and Quality of Fruit and Vegetables, Max Rubner-Institut, Karlsruhe, Germany
[7] University of Trento, Department of Physics, Bioorganic Chemistry Laboratory, Via Sommarive 14, 38123 Povo, TN, Italy

* Correspondence: fulvio.mattivi@unitn.it; Tel.: +39-0461-615-259 (F.M.)

SUPPLEMENTARY MATERIAL

Table S1. Flavan-3-ol composition of study apples as determined by UHPLC-ESI-QqQ-MS/DAD following the procedure described in [35].

Compound	µmol in 400 g of Elstar apples
Free catechin	86.57 ± 4.68
Free epicatechin	688.85 ± 29.96
Free gallocatechin	0.17 ± 0.03
Free epigallocatechin	0.00 ± 0.00
Free catechin gallate	0.18 ± 0.00
Catechin terminal units	633.01 ± 35.64
Epicatechin, terminal units	2262.56 ± 58.30
Gallocatechin, terminal units	1.19 ± 0.20
Epigallocatechin, terminal units	20.30 ± 2.83
Catechin gallate, terminal units	1.46 ± 0.23
Catechin+epicatechin + phloroglucinol	14863.06 ± 615.46
Epigallocatechin + phloroglucinol	450.69 ±45.26
Epicatechin gallate + phloroglucinol	17.66 ± 4.57
Procyanidin B1	176.35 ± 11.90
Procyanidin B2	1882.18 ± 62.13
Total flavan-3-ol monomers	563.04 ± 18.65
Total dimeric PACs	2058.53 ± 55.28
Total oligomeric PACs (terminal + superior units)	2922.50 ± 121.74
Total flavan-3-ols	5756.81 ± 154.10
Mean degree of polymerization (mDP)	8.5 ± 0.11

Table S2. Standard compounds used for PVL and PVA quantitation, their retention times, linearity range, curve equation and R^2. The last column reports the detected compounds that were quantified using the specified calibration curve.

Standard compound	RT (min)	Range	Equation	R^2	Curve used for the calibration of
5-phenyl-γ-VL-3′-sulfate	2.88	100 µm – 10 nm	46343.1x-111.7	0.997	
5-phenyl-γ-VL-3′-glucuronide	2.10	50 µm – 100 nm	2932.73x-159.2	0.994	5-phenyl-γ-VL-methoxy-glucuronide
5-(5′-hydroxyphenyl)-γ-VL-3′-sulfate	2.28	100 µm – 50 nm	20490.2x-397.5	0.997	
5-(3′-hydroxyphenyl)-γ-VL-4′-sulfate	2.66	100 µm – 5 nm	36220.5x-67.3	0.994	• 5-phenyl-γ-VL-methoxy-sulfate (1, 2)<br>• 5-phenyl-γ-VL-sulfate-glucuronide<br>• 4-hydroxy-5-(hydroxyphenyl)-VA-sulfate<br>• 4-hydroxy-5-phenyl-VA-methoxy sulfate
5-(5’-hydroxyphenyl)-γ-VL-3’-glucuronide	1.76	20 µm – 50 nm	4154.85x+9.1	0.994	• 5-(hydroxyphenyl)-y-VL-glucuronide (3′,4′ isomer)<br>• 4-hydroxy-5-(hydroxyphenyl)-VA-glucuronide

Table S3. Quantification of selected PVLs and PVAs (μmol) at different time points in urine of 11 study subjects

	5-phenyl-γ-VL-3′-sulfate					
	**0-1 H**	**1-2 H**	**2-4 H**	**4-6 H**	**6-12 H**	**12-24 H**
**S1**	0.001896	0.005282	0.000421	0.05835	2.006898	0.094276
**S2**	0.03518	0.147459	0.602618	0.972602	4.36228	2.214342
**S3**	0.02827	0.028659	0.017262	0.226868	0.873558	1.250919
**S4**	0.004746	0.000032	3.462032	1.904976	2.936	2.538965
**S5**	0.000528	0.000595	0.063616	1.083779	1.630233	0.17082
**S6**	0.000258	0.001044	0.000494	0.09581	1.147661	5.777099
**S7**	5.39E-05	0.000359	0.15984	0.223338	1.757376	1.168908
**S8**	0.005938	0.000225	0.000685	0.010384	0.18391	0.355999
**S9**	0.000292	0.004717	0.896566	0.974883	4.19083	2.539058
**S10**	0.131511	0.046062	0.117525	0.342695	12.90533	10.54295
**S11**	0.621251	0.85352	1.400177	4.855904	35.06078	10.43365

	5-(3′-hydroxyphenyl)-γ-VL-4′-sulfate					
	**0-1 H**	**1-2 H**	**2-4 H**	**4-6 H**	**6-12 H**	**12-24 H**
**S1**	0.02476	0.218044	0.875363	7.36701	69.18813	1.382005
**S2**	0.433188	0.824707	4.077125	10.26854	43.08864	9.487121
**S3**	2.389781	2.513285	2.566494	35.3891	87.89143	37.29121
**S4**	0.197995	1.179064	38.33872	33.60361	83.63832	17.58936
**S5**	0.169905	0.230921	8.083883	34.19901	57.88222	3.727891
**S6**	0.003048	0.059827	0.346346	2.269014	14.94503	22.58768
**S7**	0.006197	0.3072	3.719	4.128896	30.55488	19.07525
**S8**	0.270251	0.13424	0.338459	6.013964	63.36023	21.8509
**S9**	0.250094	0.723092	9.021644	28.72569	108.4844	44.84253
**S10**	0.561869	0.148446	1.060775	3.383349	38.68688	22.3959
**S11**	0.084806	8.47762	0.835449	2.26336	13.54982	6.195584

	5-phenyl-γ-VL-3′-glucuronide					
	**0-1 H**	**1-2 H**	**2-4 H**	**4-6 H**	**6-12 H**	**12-24 H**
**S1**	0.001964	0.002332	0.004603	0.007364	0.019516	0.012213
**S2**	0.001289	0.003191	0.003498	0.004787	0.009819	0.014361
**S3**	0.00043	0.001191	0.005155	0.003314	0.01031	0.008039
**S4**	0.001718	0.001964	3.020332	0.976824	0.23856	0.017552
**S5**	0.005769	0.006505	0.008715	0.38076	0.011722	0.00896
**S6**	0.002823	0.003559	0.005401	0.005401	0.010678	3.674319
**S7**	0.000589	0.003928	0.006812	0.005216	0.011783	0.009574
**S8**	0.00178	0.002455	0.007487	0.0027	0.01301	0.017552
**S9**	0.003191	0.003253	0.093645	0.553014	3.159665	2.46089
**S10**	0.00135	0.003314	0.006137	0.002087	2.321258	0.424995
**S11**	0.036309	0.2769	0.496619	2.922992	19.62698	2.240441

	5-(hydroxyphenyl)-γ-VL-glucuronide (3’, 4’ isomer)					
	**0-1 H**	**1-2 H**	**2-4 H**	**4-6 H**	**6-12 H**	**12-24 H**
**S1**	0.001031	0.001224	0.002416	1.40919	18.12369	0.006411
**S2**	0.032314	0.001675	0.915221	4.984805	16.04832	1.830641
**S3**	0.524528	0.451734	0.402675	22.59619	59.57633	9.613632
**S4**	0.089586	0.167208	55.3492	56.06874	60.7852	1.936506
**S5**	0.003028	0.003415	4.287868	39.34235	39.9838	0.004703
**S6**	0.001482	0.001868	0.002835	0.002835	2.451617	7.498286
**S7**	0.000309	0.002062	0.298895	0.458979	3.069216	0.989859
**S8**	0.000934	0.001289	0.00393	1.734337	70.63951	9.064842
**S9**	0.001675	0.001707	4.811635	52.34109	178.57	39.86912
**S10**	0.043115	0.00174	0.003222	0.383571	12.99443	3.183838
**S11**	0.002513	4.19602	0.004703	0.235264	2.34657	0.122741

	5-phenyl-γ-VL-methoxy-sulfate (1)					
	**0-1 H**	**1-2 H**	**2-4 H**	**4-6 H**	**6-12 H**	**12-24 H**
**S1**	0.00019	0.000226	0.036388	0.060405	0.211891	0.001183
**S2**	0.010092	0.025077	0.030156	0.045002	0.109692	0.117977
**S3**	0.006061	0.010872	0.043145	0.071688	0.211798	0.120101
**S4**	0.014827	0.01575	0.088939	0.064769	0.189576	0.146875
**S5**	0.045186	0.00063	0.071593	0.063559	0.138642	0.072828
**S6**	0.000273	0.000345	0.000523	0.041961	0.085895	0.105492
**S7**	5.71E-05	0.00038	0.055356	0.0428	0.100771	0.08612
**S8**	0.013857	0.000238	0.000725	0.023727	0.16368	0.154805
**S9**	0.000309	0.025398	0.050381	0.058743	0.180073	0.124164
**S10**	0.010747	0.000321	0.000595	0.016431	0.154535	0.154933
**S11**	0.000464	0.046526	0.070069	0.031141	0.135533	0.103944

	5-phenyl-γ-VL-methoxy-sulfate (2)					
	**0-1 H**	**1-2 H**	**2-4 H**	**4-6 H**	**6-12 H**	**12-24 H**
**S1**	0.015576	0.000226	0.037539	0.076671	0.365271	0.095734
**S2**	0.010609	0.025258	0.040604	0.081333	0.283852	0.150228
**S3**	0.008639	0.016461	0.047737	0.179906	0.594649	0.265678
**S4**	0.013559	0.015732	0.202006	0.148295	0.429664	0.188145
**S5**	0.000559	0.00063	0.106283	0.216948	0.418132	0.094239
**S6**	0.000273	0.028104	0.000523	0.045298	0.107036	0.148069
**S7**	5.71E-05	0.00038	0.053336	0.044194	0.126662	0.108861
**S8**	0.013826	0.000238	0.000725	0.031477	0.329464	0.196975
**S9**	0.024808	0.025893	0.056388	0.166272	0.664131	0.364758
**S10**	0.011477	0.025893	0.049018	0.027363	0.203094	0.187993
**S11**	0.000464	0.053128	0.000868	0.035752	0.162196	0.109163

	5-phenyl-γ-VL-methoxy-glucuronide					
	**0-1 H**	**1-2 H**	**2-4 H**	**4-6 H**	**6-12 H**	**12-24 H**
**S1**	0.017907	0.001224	0.043238	0.06711	0.559736	0.118012
**S2**	0.016287	0.027196	0.030553	0.132337	0.292064	0.119141
**S3**	0.011719	0.016302	0.002706	0.171808	0.458543	0.140894
**S4**	0.038979	0.019275	0.360696	0.207036	0.374808	0.177713
**S5**	0.003028	0.003415	0.094998	0.266055	0.346121	0.097867
**S6**	0.022848	0.001868	0.002835	0.044878	0.121204	0.104871
**S7**	0.007217	0.002062	0.06153	0.002738	0.190003	0.122581
**S8**	0.046355	0.026933	0.00393	0.060273	0.727944	0.207343
**S9**	0.026031	0.001707	0.279299	0.23473	0.97854	0.442066
**S10**	0.22628	0.079542	0.166948	0.173655	0.379391	0.378929
**S11**	0.03736	0.219008	0.004703	0.038074	0.008698	0.117187

	5-phenyl-γ-VL-sulfate-glucuronide					
	**0-1 H**	**1-2 H**	**2-4 H**	**4-6 H**	**6-12 H**	**12-24 H**
**S1**	0.001031	0.02268	0.047154	0.311391	4.984531	0.142021
**S2**	0.02851	0.03095	0.177193	0.764917	4.978764	0.847724
**S3**	0.23553	0.155001	0.12365	1.029977	6.872132	3.879614
**S4**	0.022404	0.051838	3.810523	7.499738	18.10053	4.435338
**S5**	0.050762	0.003415	0.350744	2.502551	7.160838	0.599538
**S6**	0.001482	0.035132	0.043496	0.249407	2.190234	4.348229
**S7**	0.000309	0.002062	0.160892	0.285536	2.883341	2.156298
**S8**	0.019026	0.019218	0.08228	0.359855	6.050061	3.042189
**S9**	0.026663	0.051125	0.849024	3.184713	17.86215	8.934781
**S10**	0.034613	0.033988	0.056788	0.192339	1.643909	1.133559
**S11**	0.043097	0.961322	0.094896	0.186642	1.369184	0.543123

	4-hydroxy-5-(hydroxyphenyl)-VA-sulfate					
	**0-1 H**	**1-2 H**	**2-4 H**	**4-6 H**	**6-12 H**	**12-24 H**
**S1**	0.00019	0.000226	0.000446	0.000713	4.666094	0.001183
**S2**	0.000125	0.000309	0.000339	0.000464	0.000951	0.001391
**S3**	0.182602	0.15471	0.000499	1.546209	5.576214	3.142657
**S4**	0.000166	0.00019	0.000666	4.30416	0.001902	1.830615
**S5**	0.000559	0.00063	0.000844	2.063894	0.001135	0.000868
**S6**	0.000273	0.000345	0.000523	0.000523	0.637188	1.210109
**S7**	5.71E-05	0.00038	0.00066	0.000505	0.001141	1.815021
**S8**	0.000172	0.000238	0.000725	0.000262	5.20036	2.184254
**S9**	0.010426	0.000315	0.000493	0.000499	7.163422	0.000874
**S10**	0.000131	0.000321	0.058025	0.000202	0.001605	0.001724
**S11**	0.000464	0.000476	0.000868	0.00038	0.292073	0.001266

	4-hydroxy-5-phenyl-VA-methoxy-sulfate					
	**0-1 H**	**1-2 H**	**2-4 H**	**4-6 H**	**6-12 H**	**12-24 H**
**S1**	0.00019	0.000226	0.000446	0.000713	0.001891	0.001183
**S2**	0.000125	0.000309	0.000339	0.000464	0.000951	0.001391
**S3**	0.000777	0.000115	0.000499	0.004577	0.017472	0.013919
**S4**	0.000166	0.00019	0.000666	0.000428	0.001902	0.0017
**S5**	0.000559	0.00063	0.000844	0.005187	0.001135	0.000868
**S6**	0.000273	0.000345	0.000523	0.000523	0.001034	0.001118
**S7**	5.71E-05	0.00038	0.00066	0.000505	0.001141	0.011076
**S8**	0.000172	0.000238	0.000725	0.000262	0.016218	0.0017
**S9**	0.000309	0.000315	0.000493	0.000499	0.02192	0.000874
**S10**	0.000131	0.000321	0.000595	0.000202	0.001605	0.001724
**S11**	0.000464	0.000476	0.000868	0.00038	0.001605	0.001266

	4-hydroxy-5-(hydroxyphenyl)-VA-glucuronide					
	**0-1 H**	**1-2 H**	**2-4 H**	**4-6 H**	**6-12 H**	**12-24 H**
**S1**	0.001031	0.001224	0.002416	0.003866	0.010244	0.006411
**S2**	0.000677	0.001675	0.001836	0.002513	0.005154	0.007538
**S3**	0.000226	0.005936	0.002706	0.001931	0.104034	0.091242
**S4**	0.000902	0.001031	0.003608	0.148464	0.010309	0.009213
**S5**	0.003028	0.003415	0.004575	0.002448	0.006153	0.004703
**S6**	0.001482	0.001868	0.002835	0.002835	0.19227	0.006056
**S7**	0.000309	0.002062	0.003576	0.002738	0.006185	0.005026
**S8**	0.000934	0.001289	0.017873	0.001417	0.00683	0.009213
**S9**	0.001675	0.001707	0.002674	0.002706	0.004413	0.004736
**S10**	0.000709	0.00174	0.003222	0.001095	0.008698	0.009342
**S11**	0.002513	0.002577	0.004703	0.178592	0.367875	0.418758

Table S4. Peak areas of epicatechin phase II metabolites at different time points in urine of 11 study participants

Epicatechin sulfate						
	**0-1 H**	**1-2 H**	**2-4 H**	**4-6 H**	**6-12 H**	**12-24 H**
**S1**	1326389	6729630	7627886	4545819	537949.1	115695.8
**S2**	2286928	4686064	4759275	2693558	522643.9	47355.99
**S3**	12001577	47765606	23652766	19061541	3626151	486581.6
**S4**	3381769	11963453	8665182	3894692	735901.3	225699.5
**S5**	203828.6	4514361	7777964	5027105	1117218	333298.3
**S6**	36209.58	13144646	15101821	11624780	2639002	364616
**S7**	7942834	10757225	11869435	9368591	3299.233	111671.6
**S8**	366346.4	8557358	11639119	10917464	980372.2	87007.98
**S9**	286249.8	5551614	12297950	9452829	2835509	334020.5
**S10**	1457951	6334921	13595429	14717488	878015.3	143436.4
**S11**	872249.3	6761057	10234210	8407972	1080709	218703.1

Epicatechin methoxy-sulfate						
	**0-1 H**	**1-2 H**	**2-4 H**	**4-6 H**	**6-12 H**	**12-24 H**
**S1**	264155.2	1505928	1715618	756096.5	842118.3	155791.6
**S2**	515779.8	1604495	1794357	721462.2	1040562	113782.8
**S3**	2228138	8786534	5138701	4409302	1465165	99307.92
**S4**	690347.8	2486397	2201221	1080031	751369.8	196323.9
**S5**	241809.9	1220005	2273699	1693201	329099.7	379075.3
**S6**	95482.43	3308303	4418853	2910722	687244.1	197620.7
**S7**	1669724	2199023	2670830	1726327	62038.97	139673.9
**S8**	423632.2	1720478	2936943	2295600	405058	364021.1
**S9**	418943.1	1388609	3588964	3329649	865319	352424
**S10**	447708.8	1596924	3185476	3001613	268254.5	273954.2
**S11**	245223.5	1957469	2759612	2162724	478304.9	410378.1

Epicatechin glucuronide						
	**0-1 H**	**1-2 H**	**2-4 H**	**4-6 H**	**6-12 H**	**12-24 H**
**S1**	202881.9	1629378	2359769	1499848	309913.7	77646.22
**S2**	228722.1	633382.3	743255.2	528876.8	174653.8	0
**S3**	1558598	5973485	3321729	4855303	1043087	139266.5
**S4**	399656	1849520	1877834	1034390	225865.7	52267.37
**S5**	17571.76	623071	1121546	1151800	243468.1	70097.7
**S6**	5260.954	2847977	3788255	3382646	1297641	301090.7
**S7**	668327.6	1157062	1566698	1297940	0	43100.36
**S8**	76165.32	2498802	4169011	6193930	986075	149360.9
**S9**	14363.95	1822636	5357244	5290114	2265415	211945
**S10**	350289.2	1848168	3813223	4550370	442151.6	96873.25
**S11**	151091.6	1443595	1762950	2093796	475948.4	117135.1

Epicatechin methoxy-glucuronide						
	**0-1 H**	**1-2 H**	**2-4 H**	**4-6 H**	**6-12 H**	**12-24 H**
**S1**	53325.73	187265.3	410527.9	263698.4	44379.53	3334.106
**S2**	57420.7	144792.3	174158.7	143130.9	64053.84	5234.604
**S3**	206971.1	642872.9	545616.8	606339.6	139712.8	8155.154
**S4**	79783.13	256745.1	235160.9	202879.7	49920.92	9343.942
**S5**	7575.494	75754.94	174440.7	197368.9	35440.03	2925.878
**S6**	42551.95	425519.5	732121.4	698675.9	286720.2	157462.7
**S7**	91762.97	124026.5	226577.2	226080.1	0	47443.82
**S8**	11057.8	370447.6	764561.8	953267.9	289455.2	106957.7
**S9**	12971.22	320977.4	917894.5	967904.3	445749.4	101725.8
**S10**	62764.87	269044.5	527606.6	627855.8	122608.4	32897.62
**S11**	27498.78	232632.1	371709.3	431238.5	160841	91763.81

Table S5. Composition of the Keto-Drink. Ingredients: water, rapeseed oil, pea protein, dietary fibre: corn dextrin, medium-chain triglycerides (MCT), calcium phosphate, magnesium hydroxide, chromium chloride, ferrous gluconate, potassium chloride, potassium iodate, potassium lactate, copper gluconate, manganese sulphate, sodium selenate, zinc gluconate, sodium L-ascorbate, D-biotin, cyanocobalamin, menaquinone, nicotinamide, calcium D-pantothenate, phylloquinone, folic acid, pyridoxine hydrochloride, retinyl acetate, riboflavin, DL-alpha tocopheryl acetate, cholecalciferol, vitamin E rich oil from palm fruit/annatto, choline tartrate, L-carnitine, maltodextrin, aroma, emulsifier: mono and diglycerides of edible fatty acids. Details on the composition of the Keto-Drink (neutral taste) are available online at the homepage of the manufacturer at: https://www.keto-drink.de/en/keto-drink-neutral-taste/

Nutrition Facts

**Nutrients**	**per 100 ml**	**per drink (250 ml)**
**Energy**	828 kJ	2,070 kJ
**Energy**	200 kcal	500 kcal
**Fat**	16.8 g	42 g
**of which saturated fatty acids**	4.6 g	11.5 g
**of which mono-unsaturated fatty acids**	8.5 g	21.25 g
**of which polyunsaturated fatty acids**	3.7 g	9.25 g
**Carbohydrate**	1.6 g	4 g
**of which sugars**	0.2 g	0.5 g
**Fibre**	3.1 g	7.75 g
**Protein**	7.4 g	18.5 g
**Salt**	0.23 g	0.575 g
**Vitamins**	**per 100 ml**	**per drink (250 ml)**
**Vitamin A**	174 µg	435 µg
**Vitamin D**	4 µg	10 µg
**Vitamin E (α-tocopherol equivalent)**	7.1 mg	17.75 mg
of which tocopherols	8.9 mg	22.25 mg
of which tocotrienols	10.5 mg	26.25 mg
**Vitamin K**	11.2 µg	28 µg
**Vitamin C**	8.6 mg	21.5 mg
**Thiamin**	0.2 mg	0.5 mg
**Riboflavin**	0.2 mg	0.5 mg
**Niacin (NE)**	2.6 mg	6.5 mg
**Vitamin B6**	0.3 mg	0.75 mg
**Folic acid**	44 µg	110 µg
**Vitamin B12**	0.6 µg	1.5 µg
**Biotin**	8.9 µg	22.25 µg
**Pantothenic acid**	1.2 mg	3 mg
**Minerals**	**per 100 g**	**per drink (250 ml)**
**Sodium**	92 mg	230 mg
**Potassium**	285 mg	712.5 mg
**Chloride**	130 mg	325 mg
**Calcium**	98 mg	245 mg
**Phosphorus**	92 mg	230 mg
**Magnesium**	28 mg	70 mg
**Iron**	3.4 mg	8.5 mg
**Zinc**	2.1 mg	5.25 mg
**Copper**	210 µg	525 µg
**Manganese**	0.3 mg	0.8 mg
**Selenium**	15 µg	37.5 µg
**Chromium**	9.2 µg	23 µg
**Molybdenum**	11 µg	27.5 µg
**Iodine**	48.0 µg	120 µg
**Other ingredients**	**per 100 ml**	**per drink (250 ml)**
**MCT-Fat**	3.5 g	8.75 g
**Omega-3 fatty acid**	1 g	2.5 g
**Omega-6 fatty acid**	2.6 g	6.5 g
**Choline**	81 mg	203 mg
**L-carnitine**	40 mg	100 mg
**Lactate**	1.3 g	3.25 g

Table S6. Mass spectrometry characteristic of (epi)catechin metabolites in urine samples after apple intake. For each metabolite is reported a) retention time (min), b) the theoretical formula, molecular weight (MW) and the identification level, c) ESI polarity, d) *m/z* of molecular ion and fragments with their relative intensities, e) the annotation and f) the fragments obtained in MS/MS

Metabolite	Rt (min)	Formula<br>MW<br>ID level	ESI	*m/z* (relative intensity)	annotation	MS/MS fragments
(Epi)catechin-glucuronide	4.48	$C_{21}H_{22}O_{12}$<br>466.1111<br>Level II	neg	**465.1007(100)**<br>*466.1046(11)*<br>*467.1094(1.3)*<br>487.0824(30)<br>289.0700(15)	[M-H]$^-$<br><br><br>[M-2H+Na]$^-$<br>[M-H-GLC]$^-$	**MS/MS^2 465:** 289.0706(100) [M-H-GLC]$^-$<br>327.0708(7) [M-H-$C_7H_7O_3$]$^-$<br>245.0806(5)[M-H-GLC-CO_2]$^-$<br>179.0346 (2)<br>**MS/MS^2 289:** 245.0818(100)
(Epi)catechin-sulfate	4.71	$C_{15}H_{13}O_9S$<br>369.0280<br>Level II	neg	**369.0278(100)**<br>*370.0312(18)*<br>*371.0278(9)*	[M-H]$^-$	**MS/MS^2 369:** 289.0707(100) [M-H-SULF]$^-$<br>230.9963(65) [M-H-$C_7H_5O_3$]$^-$<br>216.9801(10) [M-H-$C_8H_7O_3$]$^-$<br>245.0812(10) [M-H-SULF-CO_2]$^-$
(Epi)catechin-methoxy glucuronide	4.86	$C_{22}H_{24}O_{12}$<br>480.1268<br>Level II	neg	**479.1159(100)**<br>*480.1168(14)*<br>*481.1202(2)*	[M-H]$^-$	**MS/MS^2 479:** 303.0851(100) [M-H-GLC]<br>313.0536(50) [M-H- $C_9H_{10}O_3$]$^-$
			pos	481.1317(100)<br>*482.1351(10)*	[M+H]$^+$	
(Epi)catechin-methoxy sulfate	5.25	$C_{16}H_{16}O_9S$<br>384.0515<br>Level II	neg	**383.0438(100)**<br>*384.0469(21)*<br>*385.0436(9)*	[M-H]$^-$	**MS/MS^2 383:** 303.0863(100) [M-H-SO_3]<br>216.9807(61) [M-H-$C_9H_{10}O_3$]$^-$<br>137.0242 (38) [M-H-$C_9H_{10}O_6S$]$^-$<br>285.0750(6) [M-H-SULF-H_2O]$^-$

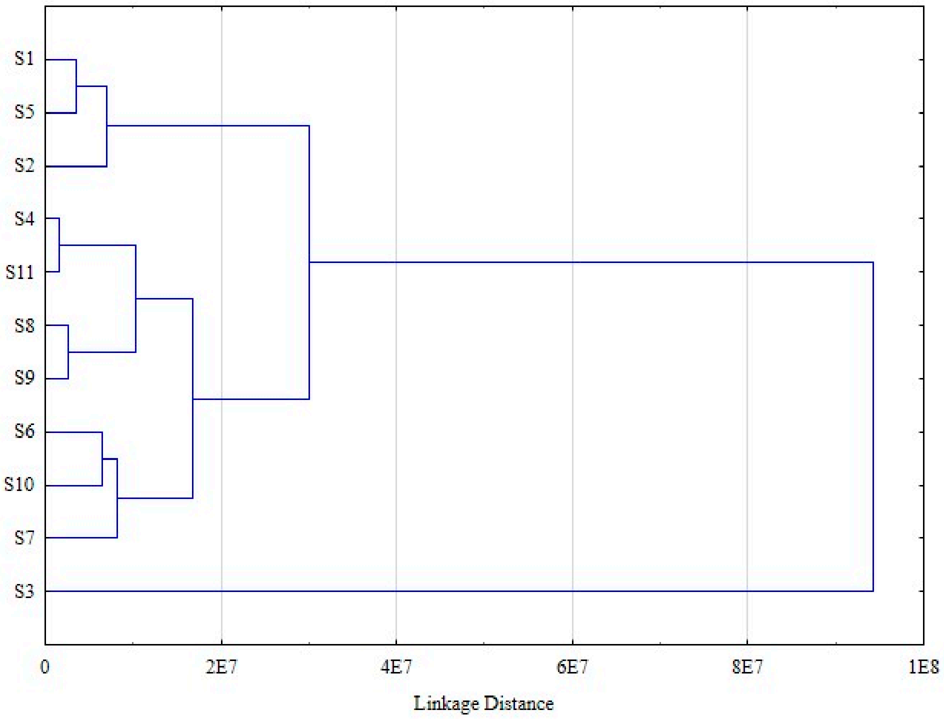

Figure S1: Cluster analysis based on percentages of excreted epicatechin phase II metabolites in the 11 subjects (complete linkage, Euclidean distance)
